# *In Situ* Homogeneous Generation of Copper Nanoparticles in Collagen-Cellulose Freeze-Dried Foams Using Natural Reduction Agents to Enhance Their Stability, Antibacterial Properties, and Cytocompatibility

**DOI:** 10.1021/acsomega.5c03661

**Published:** 2025-07-28

**Authors:** Veronika Polakova, Jana Matulova, Jana Brtnikova, Zdenka Fohlerova, Kristyna Smerkova, Jozef Kaiser, Tomas Zikmund, Petra Prochazkova, Jan Zidek, Lucy Vojtova

**Affiliations:** † Central European Institute of Technology, Brno University of Technology, Purkynova 123, 612 00 Brno, Czech Republic; ‡ Department of Chemistry and Biochemistry, Faculty of AgriSciences, Mendel University in Brno, Zemedelska 1, 613 00 Brno, Czech Republic; § Faculty of Mechanical Engineering, Brno University of Technology, Technicka 2, 616 69 Brno, Czech Republic

## Abstract

The treatment of chronic wounds remains a major challenge in regenerative medicine due to prolonged healing times, susceptibility to infection, and underlying conditions like diabetes. Incorporating bioactive and antibacterial nanoparticles (NPs) into wound dressings can significantly enhance their mechanical properties, structural integrity, and functionality, improving stability, biocompatibility, and healing efficacy. However, conventional methods of loading NPs in polymer matrices often lead to uneven distribution and localized toxicity. To overcome these limitations, we employ a novel *in situ* synthesis of copper nanoparticles (CuNPs) using an encapsulation method via the self-assembled polymerization of dopamine (DOPA) or tannic acid (TA) within collagen/carboxymethyl cellulose (Coll/CMC) 3D freeze-dried scaffolds. When CuNPs are synthesized *ex situ*, both DOPA and TA act as reducing and encapsulating agents. However, *in situ* synthesis within Coll/CMC scaffolds results in TA functioning solely as a reducing agent, while DOPA serves both as a reducing agent and, through its polymerization into polydopamine, as a stabilizing agent. The polydopamine network enhances collagen fiber adhesion to CuNPs and stabilizes them via noncovalent interactions. Notably, the DOPA-*in situ*/Cu sample exhibited prolonged enzymatic stability for up to 7 days. X-ray microcomputed tomography confirmed the homogeneous distribution of CuNPs throughout the scaffold. Biological assays demonstrated the enhanced antibacterial efficacy of DOPA/TA-*in situ*/Cu samples against *Staphylococcus aureus* and MRSA, along with cytocompatibility with 3T3 fibroblasts. Future research should explore the *in vivo* application of these scaffolds and their potential in regenerative medicine for treating infected wounds.

## Graphical abstract

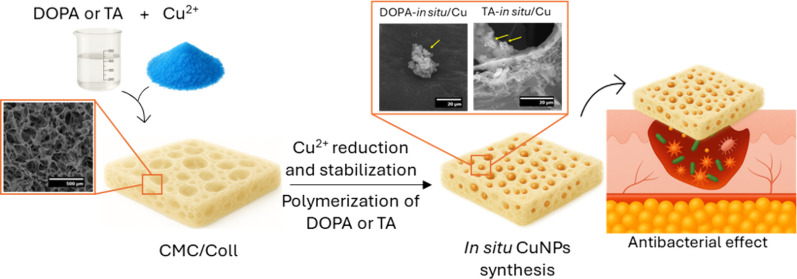

## 1 Introduction

Collagen is a biocompatible and biodegradable material that plays a crucial role in wound healing due to its structural properties and biological functions. It provides a scaffold that supports cell attachment, proliferation, migration, and differentiation while promoting hemostasis and mimicking the extracellular matrix (ECM).^1^ Collagen dressings, available in various forms such as gels, sponges, and sheets, are effective in managing chronic wounds, including pressure ulcers, diabetic ulcers, and venous ulcers.^2^ To enhance its properties, collagen has been combined with other biomaterials. For example, integrating collagen’s bioactivity with the moisture-retaining properties of carboxymethylcellulose (CMC) promotes faster and more effective wound healing, hemostasis, and controlled drug delivery.^3,4^ Additionally, incorporating CMC into collagen scaffolds improves mechanical stability and handling.^5–7^ Despite these advantages, collagen-based materials have a notable drawback: they provide an excellent substrate for bacterial growth and lack inherent antibacterial properties.^8^ Consequently, if a wound becomes infected, skin regeneration may be delayed, increasing the risk of developing a nonhealing or chronic wound.

In recent years, the incorporation of pro-healing or antibacterial agents into collagen wound dressings, foams, and hydrogels has been shown to accelerate wound healing, reduce treatment duration, and prevent infections.^4,9,10^ Biogenic antibacterial nanoparticles (NPs) play a crucial role in collagen-based scaffolds, providing both antimicrobial and pro-healing properties.^11,12^ Additionally, NPs have been shown to complement antibiotics, offering antimicrobial effects that are particularly promising for combating multidrug-resistant strains and biofilms.^12^

While various NPs, such as selenium, iron oxide, and zinc oxide based, have demonstrated potent antibacterial properties, copper NPs (CuNPs) were specifically chosen for their unique combination of antibacterial efficacy, biocompatibility, and ability to promote tissue repair.^12,13^ Copper additives within the collagen matrix are particularly promising due to their association with the copper-dependent enzyme lysyl oxidase, which catalyzes the covalent cross-linking that stabilizes collagen and elastin fibers. As a result, lysyl oxidase plays a crucial role in the morphogenesis and regenerative capacity of connective tissues, including those in the skeleton, respiratory tract, and cardiovascular system. The bioactivity of copper in tissue regeneration is further enhanced by the antibacterial properties of CuNPs. These NPs exhibit broad-spectrum antibacterial activity against both Gram-positive and Gram-negative bacteria, including antibiotic-resistant strains.^14–16^ This makes them a promising alternative to conventional antibiotics, which often lose effectiveness due to the emergence of resistant bacterial strains. The antibacterial potency of CuNPs is largely attributed to their ability to generate reactive oxygen species (ROS), allowing them to effectively target multidrug-resistant strains and disrupt biofilms.^17^

On the other hand, at higher concentrations, CuNPs can exhibit cytotoxicity.^16,18,19^ To mitigate this issue and regulate the release profile of NPs from the scaffold, encapsulation can be employed as a potential solution. Various encapsulation techniques have been explored, with chelation emerging as a particularly effective approach for stabilizing metal NPs.^20^ In this study, catechol moieties from dopamine (DOPA) and tannic acid (TA) are utilized.^21,22^ Dopamine, a neurotransmitter naturally present in the human body, undergoes self-assembly into polymers upon oxidation and has a unique ability to adhere to a wide range of surfaces.^23–25^ Similarly, tannic acid, a natural polyphenol found in plants, possesses antioxidant properties, forms complex polymers, and can adhere to and precipitate proteins on various surfaces.^26,27^

Traditional methods of incorporating NPs often lead to aggregation and uneven distribution, resulting in localized high concentrations that can compromise the scaffold’s biocompatibility and effectiveness.^28^ ^,^ ^29^ *In situ* NP synthesis within scaffolds offers a solution by ensuring a homogeneous distribution throughout the matrix, which is crucial for maintaining consistent functionality while minimizing aggregation and localized toxicity.^30^ ^,^ ^31^

In this study, an encapsulation method based on the self-assembly polymerization of TA/DOPA is utilized for the *in situ* synthesis and stabilization of CuNPs on a freeze-dried Coll/CMC scaffold. The primary objective is to achieve a uniform nanoparticle distribution within the scaffold while ensuring controlled copper release. The synthesized CuNPs are characterized in terms of size, zeta potential, and release kinetics. The resulting Coll/CMC porous matrix, with CuNPs stabilized by TA/DOPA, is analyzed using various physicochemical techniques, including X-ray microcomputed tomography, scanning electron microscopy (SEM), and stability assays, alongside antibacterial and cytotoxicity evaluations.

## 2 Materials and Methods

### 2.1 Materials

Bovine collagen type I was purchased from Collado, s.r.o., Czech Republic as a freeze-dried powder. Hydrophilic carboxymethyl cellulose, sodium salt (CMC) (*M*_*w*_ = 250,000, DS = 0.7) was purchased from Acros Organics, France. Copper­(II) sulfate pentahydrate g.r., tannin g.r. (TA), and ammonium hydroxide 25% g.r. were purchased from Carl Roth, Czech Republic. Dopamine hydrochloride g.r. (DOPA), Calcium chloride dihydrate, potassium chloride ≥ 99.0%, N-(3-(dimethylamino)­propyl)-N′-ethylcarbodiimide hydrochloride g.r., N-hydroxysuccinimide g.r., and Collagenase from *Clostridium histolyticum* ≥ 125 CDU/mg were purchased from Sigma-Aldrich, Germany. Disodium hydrogen phosphate dodecahydrate g.r., and sodium chloride g.r. were purchased from Lach-Ner, Czech Republic. Ammonium hydrogen carbonate g.r., and magnesium chloride hexahydrate g.r. were purchased from PENTA, Czech Republic. HI95747 01 Copper LR reagent was purchased from Hanna Instruments Czech, Czech Republic. LCW 902 Crack-Set was purchased from HACH LANGE, Czech Republic. All experiments were performed using ultrapure water type II prepared according to ISO 3696.

### 2.2 Preparation of Coll/CMC Scaffold

Collagen/carboxymethyl cellulose (Coll/CMC) solution was prepared by weighing both materials to obtain a final concentration of 1% (0.95% w/v collagen with 0.05% w/v CMC). Prior to mixing, CMC was allowed to swell in one-quarter of the final volume of ultrapure water. Collagen was similarly hydrated in half of the final volume of ultrapure water at 4 °C for 60 min. The swollen collagen was then disintegrated using a digital disperser at 6,000 rpm under continuous cooling. After 2.5 min, the swollen CMC was added to the mixture. The remaining ultrapure water was used to rinse residual material from the beakers and was subsequently added to the solution. The mixture was further homogenized for an additional 2.5 min. The resulting material was distributed onto a well plate and freeze-dried at −35 °C, 15 Pa for 48 h using a freeze-dryer (Martin Christ, Epsilon 2-10D LSCPlus, Osterode am Hartz, Germany).

### 2.3 *Ex Situ* Synthesis and Encapsulation of CuNPs

CuNPs encapsulated by TA and CuNPs encapsulated by DOPA were synthesized by preparing a solution of the CuNP precursor, CuSO_4_·5H_2_O, along with stabilizing agents TA or DOPA, both diluted in ultrapure water. The final concentration of the copper and catechol moieties was adjusted to 2 mg/mL. The solution was mixed on a magnetic stirrer at 250 rpm for 4 h. Subsequently, the pH was adjusted to 9 using 0.1 M NH_4_HCO_3_. The stirring process continued for an additional 20 h. Afterward, the solution was transferred to Eppendorf tubes and centrifuged at 200 rpm for 20 min to remove nanoparticle agglomerates. The supernatant was then subjected to further centrifugation at 4200 rpm for 20 min. The precipitate obtained was retained and washed by adding 3 mL of ultrapure water, followed by centrifugation at 4200 rpm for 20 min. This washing step was repeated twice to ensure purity. Finally, the nanoparticles were frozen and lyophilized. Nanoparticle size was characterized using scanning transmission electron microscopy (STEM, MIRA3, Tescan, Czech Republic) and dynamic light scattering, together with measuring zeta potential (DLS, ZetaSizer Ultra, Malvern Panalytical, United Kingdom).

### 2.4 *In Situ* Synthesis of CuNPs

The CuNPs prepared *in situ*, while utilizing DOPA (DOPA-*in situ*/Cu) or TA (TA-*in situ*/Cu) were synthesized directly onto the collagen scaffold. Copper­(II) sulfate pentahydrate was weighed to achieve a concentration of 0.1% (w/v), while DOPA or TA were each weighed to a concentration of 0.2% (w/v). Both components were dissolved in ultrapure water and stirred at 250 rpm for 4 h on a magnetic stirrer. To initiate the nanoparticle synthesis, the pH was adjusted to 9 using 0.1 M ammonium bicarbonate solution. Following this, 0.5 mL of this mixture was immediately applied to the lyophilized Coll/CMC scaffold. Nanoparticle synthesis and encapsulation proceeded directly within the scaffold for 24 h. Afterwards, the samples were thoroughly washed with ultrapure water to remove any unreacted substances. Finally, the samples were frozen and lyophilized.

### 2.5 Scaffold Characterization

#### 2.5.1 Scanning Electron Microscopy and Porosity Analysis

The structure and morphology of Coll/CMC scaffolds, both with and without CuNPs were analyzed using SEM. All samples were frozen in liquid nitrogen and then halved with a surgical blade. The sample cross-section was sputtered with a 10 nm gold layer to enhance conductivity. Images were acquired at magnifications ranging from 150× to 10,000× using secondary electron detectors and backscattered electrons. The porosity of each sample was evaluated from the acquired SEM micrographs using ImageJ software. From each sample, the diameters of 100 pores were measured, and the average pore diameter was calculated for each sample.

#### 2.5.2 X-ray Microcomputed Tomography

Freeze-dried samples were prepared for X-ray microcomputed tomography (μ-CT) analysis by freezing them in liquid nitrogen to preserve their structure. Each sample was then cut in half, and the halves were stacked on top of each other within the measurement tube, allowing three samples to be analyzed simultaneously in a single measurement. The 3D structure of the scaffolds was analyzed by μ-CT (GE Phoenix v|tome|x L240 system, Baker Hughes Digital Solutions GmbH, Wunstorf). The scans were performed with a Nanofocus X-ray tube with 180 kV/15W and a high-contrast flat panel dynamic detector 41|100 with 4000 × 4000 pixels and a pixel size of 100 × 100 μm. A total of 2100 X-ray projections were acquired with an exposure time of 400 ms. The acceleration voltage and X-ray currents utilized were 60 kV and 320 μA, respectively. Tomographic reconstruction was performed using the GE phoenix datos|x 2.0 software (Baker Hughes, Germany), the reconstructed tomographic data had a final voxel size of 5 μm. Image processing, visualization, and grayscale analysis were performed in VGStudio MAX 2024.3 software (Volume Graphics GmbH, Germany). Material segmentation was carried out with the Paint&Segment AI module.

#### 2.5.3 Measurement of Scaffold Volume

The volume of the scaffolds containing TA/DOPA with or without CuNPs, prepared via *in situ* synthesis (n = 3), along with pure Coll/CMC scaffolds serving as controls, was measured using a metric Vernier caliper. This measurement aimed to assess the impact of the *in situ* synthesis on the scaffold structure. The volume of each cylindrical sample was determined by calculating its diameter (d) and height (h) using the formula for the volume of a cylinder, as shown in Equation 1:

$$V=\pi {\left(\frac{d}{2}\right)}^{2}h\;\left[{mm}^{3}\right]$$ 1

#### 2.5.4 Swelling Properties

The swelling properties of all scaffolds with or without CuNPs (n = 4) were evaluated using the weighting method. The samples were put into glass vials and weighed in the dry state. Then the samples were swollen in Dulbecco’s phosphate-buffered saline (dPBS) at pH 7.4. The swelling kinetics were measured from 1 to 120 min. The swelling ratios were calculated according to the following Equation 2:

$$Swelling\;ratio=\frac{W_{S}-W_{0}}{W_{0}}\left[-\right]$$ 2

where *W*_S_ is the weight of the sample at time *t*, and *W*_0_ is the weight of the sample at the initial time.

#### 2.5.5 Hydrolytic and Enzymatic Stability

The hydrolytic stability of the freeze-dried samples was assessed using the weighting method by incubating samples in 2 mL of dPBS at pH 7.4 and 37 °C. The enzymatic stability of freeze-dried samples was evaluated in the presence of collagenase (10 CDU/mL) in dPBS and pH 7.4. Briefly, the samples were swollen in dPBS for 120 min. After this, the medium was replaced with 2 mL of diluted collagenase, and the samples were incubated at 37 °C. The samples were weighed at 1 to 168 h, and the stability was calculated according to Equation 3:

$$De\mathrm{grad}ation=\frac{W_{t}}{W_{S}}\times 100\;\left[\%\right]$$ 3

where *W*_*t*_ is the weight of the degraded sample at time *t*, and *W*_S_ is the weight of the sample at 60 min.

#### 2.5.6 Copper Release

The concentration of copper released from CuNPs encapsulated by DOPA or TA, as well as from Coll/CMC scaffolds with *in situ*-prepared CuNPs, was determined using UV–Vis spectrophotometry. For comparison, an additional set of samples (DOPA-coating/Cu and TA-coating/Cu) was prepared to evaluate copper release. These samples were Coll/CMC coated with *ex situ* prepared CuNPs encapsulated TA/DOPA. The Coll/CMC scaffold was prepared as described in Section 2.2. Scaffolds were cross-linked using a 0.025 M solution of N-(3-(Dimethylamino)­propyl)-N′-ethylcarbodiimide hydrochloride (EDC) in a 2:1 molar ratio with *N*-hydroxysuccinimide (NHS) in ethanol for 1 h. Following cross-linking, the scaffolds were thoroughly washed with a 0.1 M aqueous solution of Na_2_HPO_4_, with the washing solution replaced three times at 30 min intervals. The same washing process was repeated using ultrapure water. For the final step, the last water wash was replaced with a DOPA/TA-encapsulated CuNPs solution diluted in ultrapure water to a concentration of 0.45 mg/mL. The prepared samples were then frozen and freeze-dried at −35 °C and 15 Pa for 48 h using a freeze-dryer (Martin Christ, Epsilon 2-10D LSCPlus, Osterode am Harz, Germany).

The calibration curve was constructed using the standard solutions (0.01 to 5 mg/L) prepared by dissolution of copper­(II) sulfate pentahydrate in ultrapure water, and collagenase in dPBS (10 CDU/mL). The copper detection was performed using HI95747–01 copper LR reagent kit at 560 nm as recommended by the manufacturer. Briefly, the sample was leached in 2 mL of collagenase solution in dPBS (pH 7.4) at 37 °C. At defined time intervals (up to 120 h), the collagenase solution was replaced with a fresh collagenase solution, and 8 mL of ultrapure water with HI95747–01 Copper LR reagent was added to each eluate to quantify the copper release over time. After 120 h, nanoparticles were completely released from the scaffold using LCW 902 Crack-Set and the copper concentrations were subtracted from the calibration curve. The kinetic rate constant of copper release was determined by nonlinear curve fitting and Hill function using MATLAB version 24.2.0.2712019 (R2024b), Mathworks, Natick, Massachusetts, USA. The Hill equation has three parameters (Equation is presented in Supporting Information material Figures S2–5): *V*_max_, maximum released drug amount; *K*, half-time: time at which half of the nanoparticles is released; *n*, exponent determines the type of the process: −0.5, Fickian diffusion, 0.5–0.89, non-Fickian diffusion; 0.89–1, first order kinetics.

### 2.6 Antibacterial Testing

The antibacterial activity of scaffolds with *in situ*-prepared CuNPs (1 and 5 μg/mL) was tested against Gram-negative *Escherichia coli* (CCM 3954), Gram-positive *Staphylococcus aureus* (CCM 4223) and Methicillin-resistant *Staphylococcus aureus* (MRSA, CCM 7110), obtained from the Czech Collection of Microorganisms (Brno, Czech Republic). These bacterial strains were incubated on 5% Columbia blood agar (LMS, Czech Republic) at 37 °C overnight. The scaffold was placed into the tube containing 2 mL of collagenase in ultrapure water (10 CDU/mL) and 2 mL of double-concentrated Mueller-Hinton broth (Oxoid, UK) with ∼10^6^ CFU/mL of specific bacteria. Scaffolds without copper were used as controls. The scaffolds with bacteria were incubated at 37 °C under gentle shaking conditions for 24 h. After incubation, the eluate was serially diluted, and 100 μL of inoculum was plated on the Mueller-Hinton agar plates (Oxoid, UK). The plates were incubated at 37 °C for 24 h, and the number of colonies was counted and expressed as colony-forming units per milliliter (CFU/mL). All tests were performed in duplicate.

### 2.7 Cytotoxicity Assay

The extract test was used to evaluate the cytotoxic effect of the scaffolds containing *in situ* prepared CuNPs (1 and 5 μg/mL) on 3T3 fibroblast cells (modified protocol of ISO 10993–5). 3T3 fibroblasts were seeded in a 96-well plate (5 × 10^4^ cells/well) and cultured in DMEM medium containing 10% FBS and 1% penicillin-streptomycin (PS) at 37 °C with 95% humidity and 5% CO_2_ overnight. To prepare the sample extract, 0.05 g of scaffold was incubated in 1 mL of complete DMEM at 37 °C for 24 h. The cytotoxicity of the extracts was assessed using an XTT viability assay, as recommended by the manufacturer. Briefly, the culture medium was removed from the 96-well plate and replaced with 100 μL of the sample extract. After 24 h of incubation, the extract was removed, and the cells were rinsed twice with PBS. Then, 100 μL of DMEM and 50 μL of XTT were added to the cells and incubated for 3 h at 37 °C. The absorbance was measured at 480 nm using a microplate UV-Vis spectrophotometer.^32^ All tests were performed in triplicate, and pure Coll/CMC was used as the control sample.

### 2.8 Statistical Analysis

Statistical analysis of the obtained results was performed using OriginPro 2024 software. Tukey’s test was employed for pairwise posthoc comparisons, with significance levels set at 0.001, 0.01, and 0.05. Cytotoxicity testing data were analyzed using Student’s *t*-test at a 95% confidence level. Results are presented as mean ± standard error (n = 3).

## 3 Results and Discussion

### 3.1 Characterization of Encapsulated CuNPs

The encapsulation of CuNPs is achieved through the chelation of copper­(II) ions by chelating agents, specifically DOPA and TA.^33,34^ This mechanism facilitates the formation of stable polymeric capsules around the nanoparticles. To demonstrate the successful synthesis of CuNPs encapsulated either by DOPA or by TA, samples were characterized using STEM and DLS methods. The analysis of the nanoparticle size within the DOPA/TA capsules using ImageJ software on STEM micrograph revealed an average diameter of 20 ± 6 nm for CuNPs encapsulated by DOPA (Figure 1A) and 26 ± 6 nm for CuNPs encapsulated by TA (Figure 1B). The DLS measurements revealed an average hydrodynamic diameter of 384.9 ± 76.7 nm for CuNPs encapsulated by DOPA (Figure 1C) and 278.1 ± 157.0 nm for CuNPs encapsulated by TA (Figure 1D). This suggests that the DLS data primarily captured the size of the encapsulating TA structure rather than the nanoparticles themselves. The encapsulation properties of TA/DOPA polymeric capsules differ slightly due to variations in nanoparticle size. For comparison with DLS measurements, the size of the capsules was determined from STEM images. CuNPs stabilized by TA showed an average diameter of 165.1 ± 69.1 nm, while those stabilized in DOPA measured 138.8 ± 32.8 nm. The smaller average sizes observed by STEM can be attributed to the sample drying process required for imaging. For CuNPs encapsulated by TA capsule with an average diameter of 278.1 nm, a single capsule can encapsulate approximately 1.22 × 10^3^ nanoparticles. In comparison, CuNPs encapsulated by DOPA capsules, with a bigger average diameter of 384.9 nm, allow for slightly more nanoparticles per capsule, with an estimated 7.11 × 10^3^ nanoparticles per capsule. Details of the calculation for the average number of encapsulated particles per capsule are provided in Supporting Information, Section 1. The calculation of the average number of encapsulated particles in a capsule. The differences between DLS and SEM results might also be partially influenced by sample preparation methods. For SEM, the sample is dried, whereas in DLS, the nanoparticles are measured in an aqueous solution, which may promote CuNPs agglomeration, leading to an increase in the measured size.

**Figure 1:**
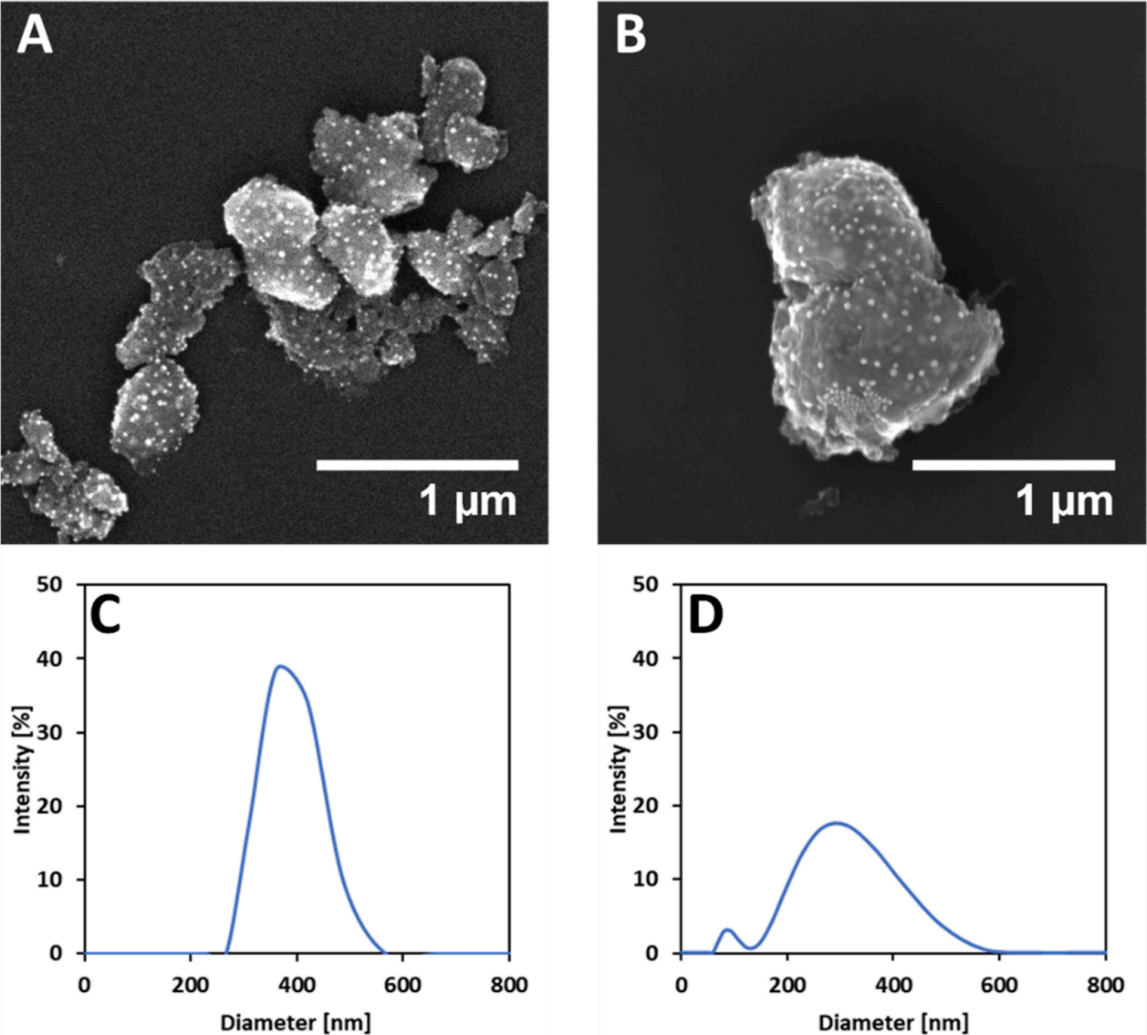
STEM micrographs of CuNPs encapsulated by DOPA (A) and CuNPs encapsulated by TA (B). DLS size distribution by intensity of CuNPs encapsulated by DOPA capsules (C) and CuNPs encapsulated by TA capsules (D).

The zeta potential of CuNPs encapsulated by DOPA and TA was measured at 25 °C immediately after reaction initiation (pH adjustment) and after 24 h to assess particle stability. Initially, the zeta potential of CuNPs encapsulated by TA was −39.4 ± 0.9 (SD) mV (mean absolute deviation = 0.7 mV), while that of CuNPs encapsulated by DOPA was −17.6 ± 1.1 (SD) mV (mean absolute deviation = 0.9 mV). The negative zeta potential values indicate the presence of hydroxyl and other functional groups on the particle surfaces due to dispersion in water. These surface charges result in electrostatic repulsion, contributing to the stability of the nanoparticles by preventing aggregation.^35^ A higher absolute zeta potential value of −39.4 ± 0.9 mV for CuNPs encapsulated by TA suggests strong electrostatic repulsion, implying good stability. In contrast, the lower absolute value of −17.6 ± 1.1 mV for CuNPs encapsulated by DOPA indicates weaker repulsive forces, which may allow slight particle agglomeration. After 24 h, zeta potential values shifted significantly, with CuNPs encapsulated by DOPA reaching −38.3 ± 6.8 (SD) mV (mean absolute deviation = 5.0 mV). This change suggests the completion of the particle encapsulation process by DOPA polymerization and capsule formation. Zeta potential of CuNPs encapsulated by TA remained relatively unchanged, shifting only slightly to −39.1 ± 4.7 (SD) mV (mean absolute deviation = 3.5 mV) after 24 h.

### 3.2 The Structure of Coll/CMC Scaffold with TA/DOPA-Stabilized CuNPs

The SEM analysis provided detailed insights into the inner structure of the scaffolds and the distribution of incorporated CuNPs (Figure 2). When comparing these samples, it was evident that the presence of CuNPs influenced the scaffold architecture. Specifically, in samples containing CuNPs, the collagen fibers appeared more melted or interconnected. In the case of DOPA-*in situ*/Cu, a melting-like deformation of collagen fibers was observed. Additionally, in all samples, nanoparticles tend to form small agglomerates within the scaffolds. This agglomeration may have resulted from the *in situ* oxidation of TA and oxidation and polymerization of DOPA, during which nanoparticles could have adhered together. The average pore diameter of each sample was evaluated using ImageJ software. The largest pores were observed in the Coll/CMC sample (99.56 ± 40.44 μm). The DOPA-*in situ* sample exhibited an average pore diameter of 68.31 ± 28.81 μm, while the DOPA-*in situ*/Cu sample showed a reduced pore size of 47.42 ± 18.34 μm. Similarly, the TA-*in situ* sample had an average pore diameter of 90.19 ± 40.57 μm, whereas the TA-*in situ*/Cu sample measured 53.51 ± 29.12 μm. Given that dopamine is known for its adhesive properties, it is likely that this contributed to the aggregation of nanoparticles and the observed structural changes. The adhesion effect might have also altered the pore size by causing polymerized structures to bind collagen fibers more tightly. Similar adhesive behavior of dopamine has been observed in other studies.^36,37^

**Figure 2:**
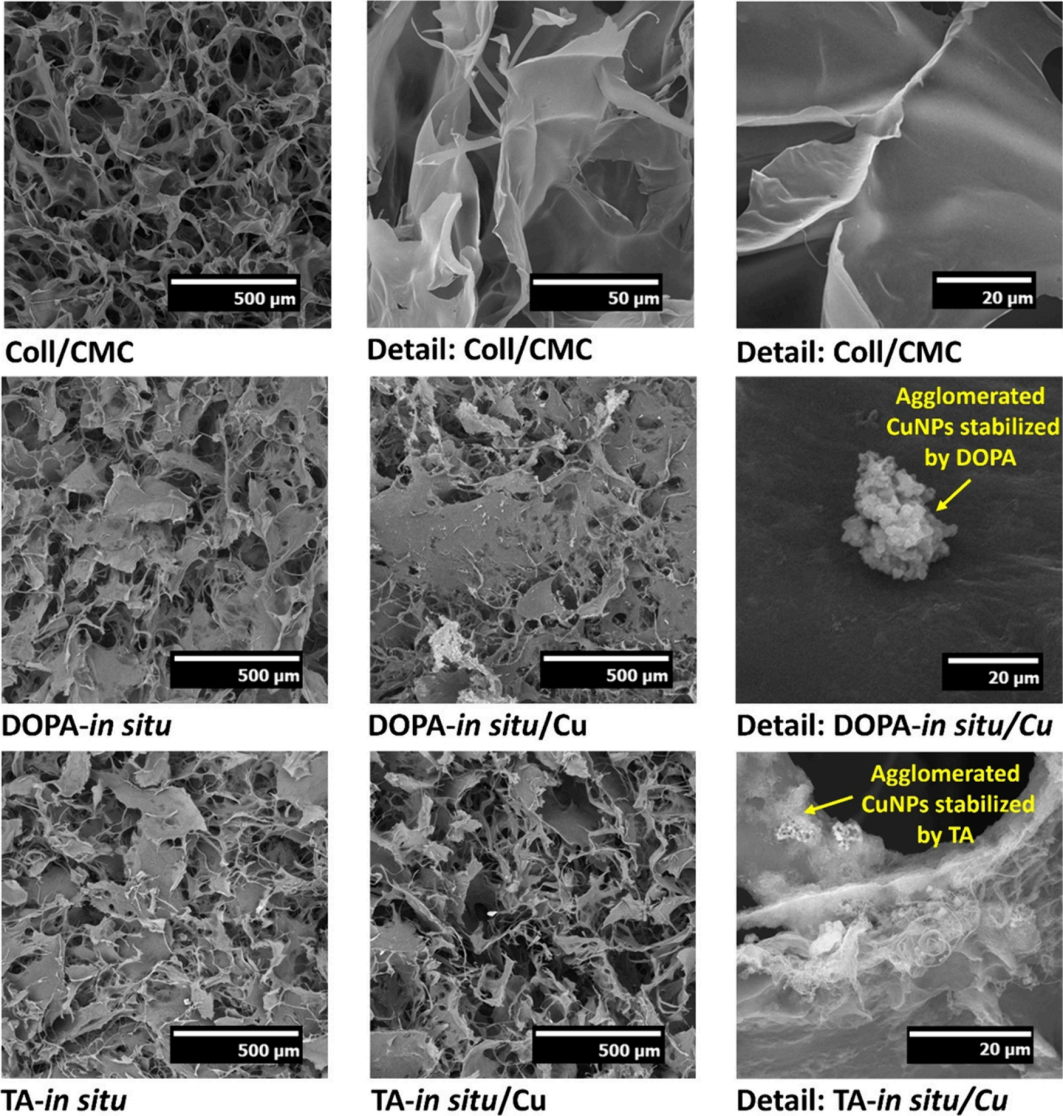
Scanning electron micrographs of samples prepared by the *in situ* method with higher magnification images that show contrasting copper in detail.

In our system, dopamine does not act as a surfactant to stabilize individual copper nanoparticles. Instead, it promotes the formation of capsule-like structures due to its unique oxidative self-polymerization behavior and the mechanisms of polydopamine capsule formation. PDA is a biopolymer with strong self-assembly and universal adhesion capabilities.^38^ ^,^ ^39^ Upon exposure to mildly alkaline conditions, dopamine rapidly undergoes oxidative polymerization, forming a net-like polydopamine network rich in catechol and amine groups. This network serves as a stable scaffold, capable of chelating metal nanoparticles (CuNPs) via coordination bonds with metal ions. Importantly, dopamine simultaneously acts as a reducing agent, reducing Cu^2+^ ions to metallic CuNPs while polymerizing into the polydopamine matrix. This dual functionality facilitates the controlled formation and embedding of CuNPs within the polydopamine structure, enhancing their stabilization and distribution. The rapid polymerization process limits the presence of dopamine in its monomeric amphiphilic form, thus preventing surfactant-like stabilization of individual nanoparticles. Instead, polydopamine forms robust capsules or coatings around nanoparticles through surface deposition and polymeric cross-linking mechanisms.^40–42^

Next, we aimed to leverage the increased contrast provided by copper nanoparticles to demonstrate their even distribution within the Coll/CMC scaffold using μ-CT. A relevant study by Zidek et al. (2016) demonstrated the utility of this technique for visualizing inorganic nanoparticles in collagen-based freeze-dried foams.^43^

The reconstructed 3D models, depicted in Figure 3, revealed significant improvements in sample visibility and contrast with the incorporation of CuNPs. Pure Coll/CMC scaffolds showed low contrast due to the low X-ray attenuation of their organic components, making the internal structure difficult to resolve. The addition of dopamine (DOPA-*in situ*) or tannic acid (TA-*in situ*) as polymeric capsules increased the density of the scaffold and thus slightly improved visibility of the internal structure, although significant uncertain regions remained. The presence of CuNPs, encapsulated within DOPA or TA capsules (DOPA-*in situ*/Cu, TA-*in situ*/Cu), further increased the contrast, allowing for precise visualization of the internal scaffold structure.

**Figure 3:**
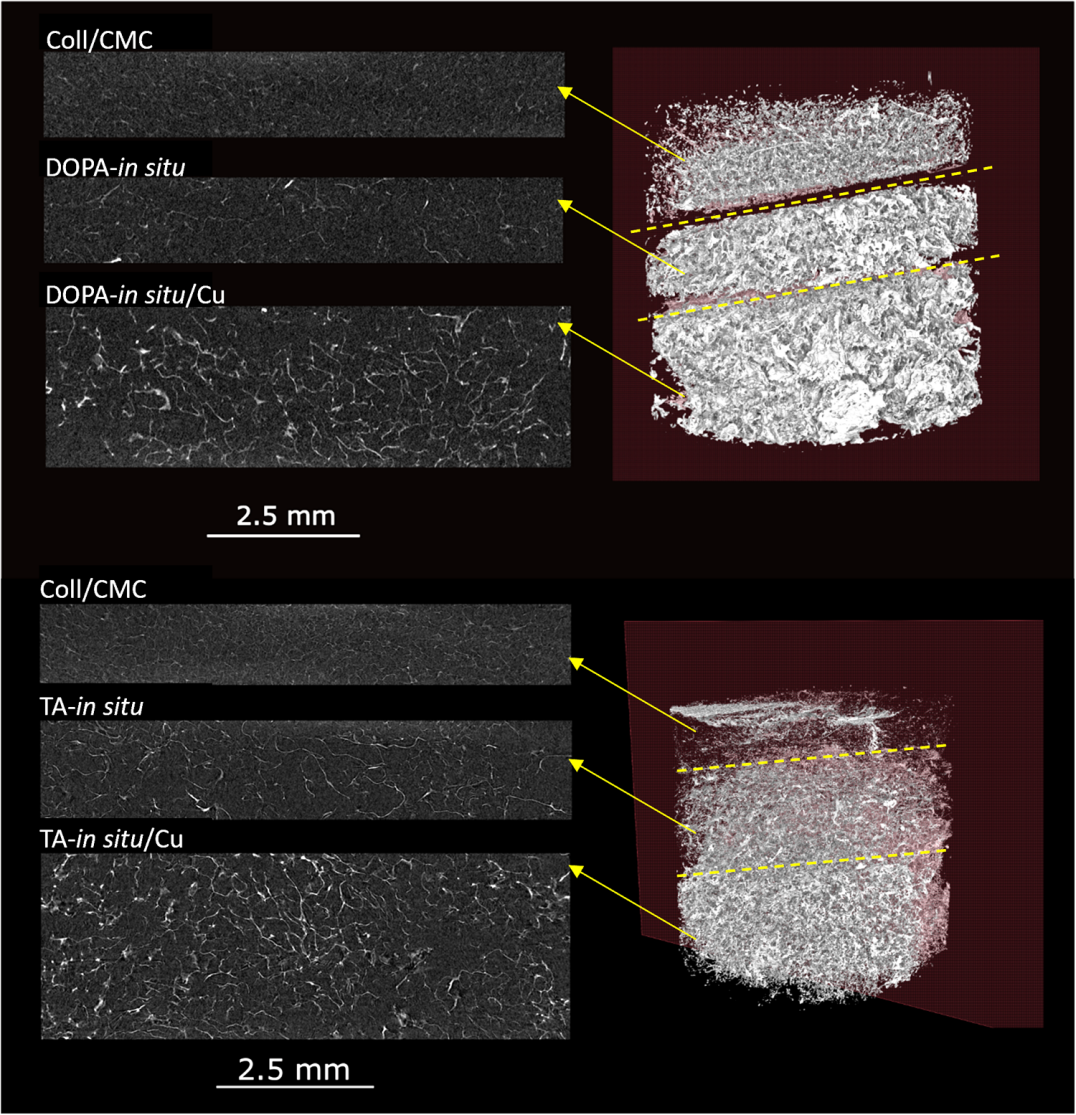
Frontal cross sections of samples. Pure Coll/CMC scaffold showing limited contrast due to the absence of elements with higher X-ray attenuation. DOPA-*in situ* and TA-*in situ* scaffolds with polymer capsules, exhibiting moderate contrast. DOPA-*in situ*/Cu and TA-*in situ*/Cu scaffolds demonstrating significantly enhanced contrast and uniform distribution of nanoparticles throughout the scaffold volume.

Frontal and transverse cross sections, depicted in Figure 3 and Figure 4, demonstrated that CuNPs were evenly distributed throughout the entire scaffold volume for both DOPA-*in situ*/Cu, and TA-*in situ*/Cu samples. No large, localized agglomeration or clustering of nanoparticles was observed, indicating the success of the *in situ* synthesis method in achieving homogeneity. The even distribution of CuNPs was further supported by the consistent grayscale intensity across the scanned regions, representing uniform nanoparticle integration within the collagen matrix. The contrast-to-noise ratio (CNR) analysis was used to evaluate the image contrast between different types of scaffold materials and background noise. For samples with the addition of dopamine, DOPA-in situ/Cu exhibited the highest CNR (1.98), indicating the best contrast and distinguishability of the scaffold. In contrast, DOPA-in situ material showed the lowest CNR (0.88), while the pure Coll/CMC scaffold had a CNR value of 0.99. Similarly, for the tannic acid samples, TA-in situ/Cu showed the highest CNR (1.89), TA-in situ material exhibited the lowest CNR (1.17), and pure Coll/CMC scaffold had a CNR value of 1.45.

**Figure 4:**
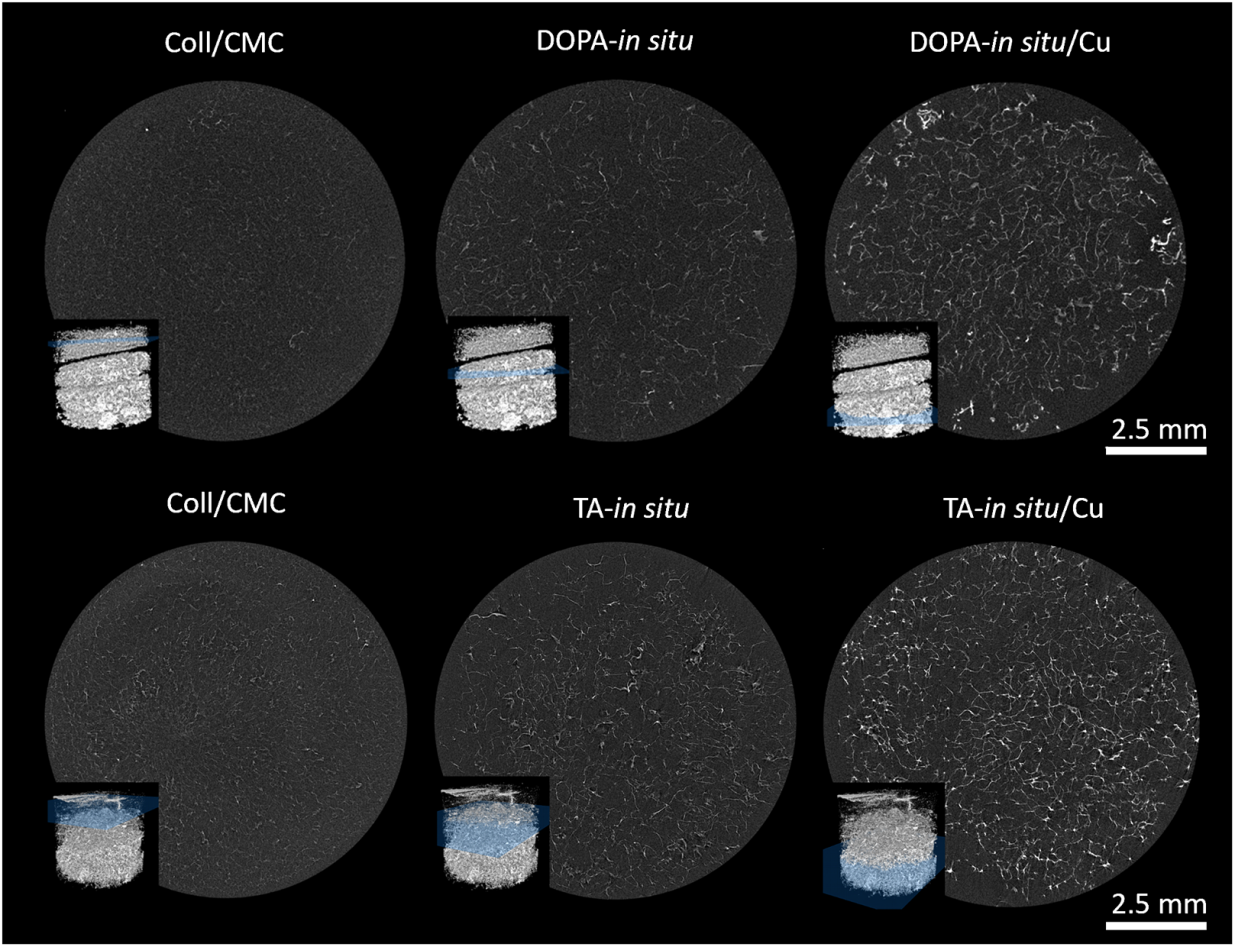
Transversal cross sections of samples. Pure Coll/CMC scaffold showing limited contrast due to the absence of elements with higher X-ray attenuation. DOPA-*in situ* and TA-*in situ* scaffolds with polymer capsules, exhibiting moderate contrast. DOPA-*in situ*/Cu and TA-*in situ/*Cu scaffolds demonstrate significantly enhanced contrast and uniform distribution of nanoparticles throughout the scaffold volume.

To evaluate the impact of TA/DOPA with or without CuNPs on Coll/CMC scaffolds, the sizes of the samples were measured by volumetric method. Real shapes of all samples were approximated to a cylinder (Figure 5B), and their volumes were calculated and compared. Significant differences were found in between sample sizes of pure Coll/CMC scaffold (400 ± 37 mm^3^) and all Coll/CMC with CuNPs (Figure 2A). The smallest sample volume was observed for the DOPA-*in situ*/Cu scaffold (155 ± 23 mm^3^) even when compared to other samples: the DOPA-*in situ*/Cu sample (250 ± 87 mm^3^), TA-*in situ* sample (250 ± 56 mm^3^) and TA-*in situ*/Cu sample (220 ± 22 mm^3^). These variations in volume size may be attributed to the influence of CuNPs on collagen rearrangement within the Coll/CMC scaffold, as well as the adhesive properties of DOPA and TA.

**Figure 5:**
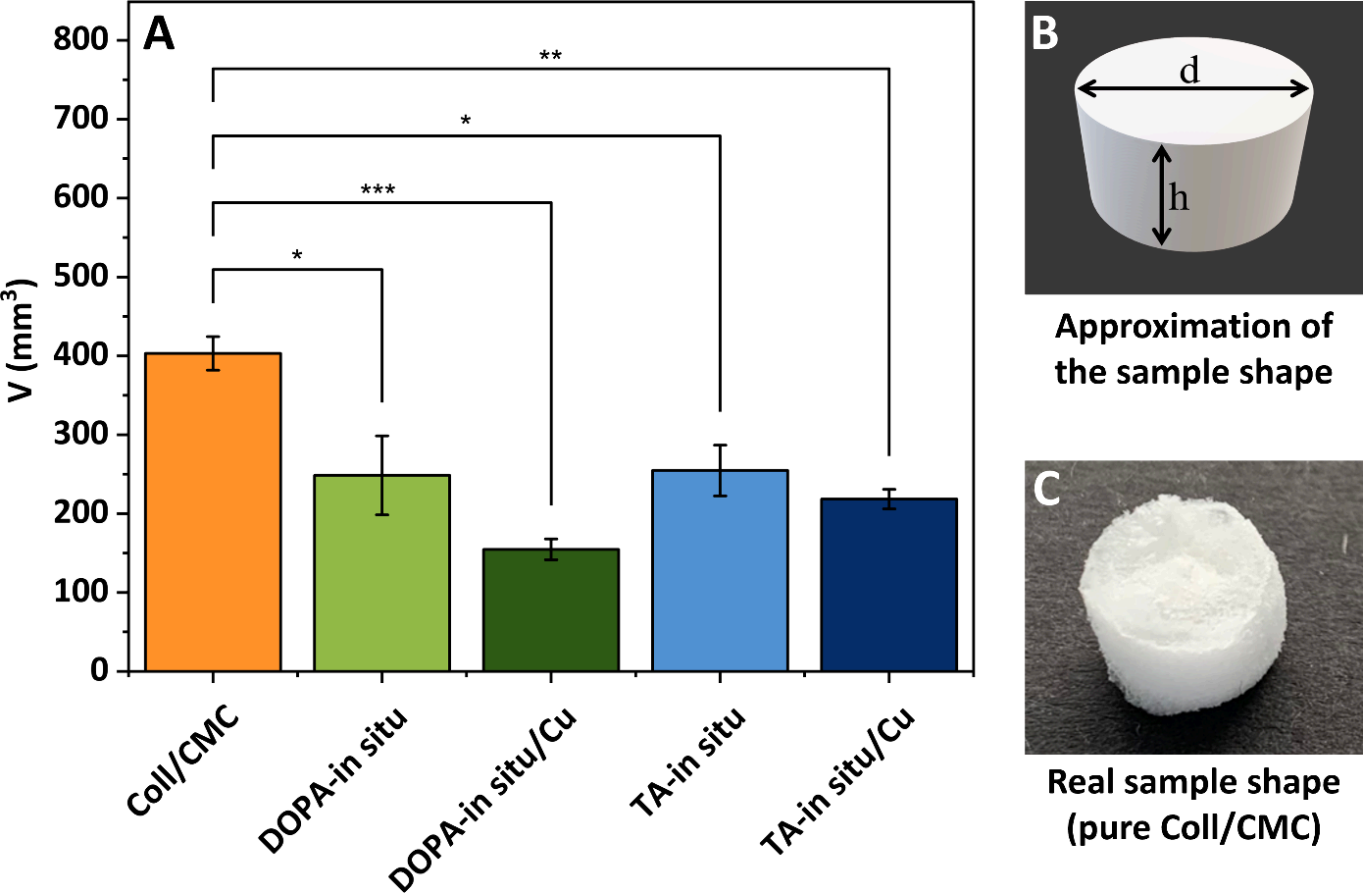
(A) Comparison of average sample volume of the pure Coll/CMC and *in situ* prepared samples with or without CuNPs. *P* values reaching statistical significance (*p* < 0.001) were marked ∗∗∗. *P* values reaching statistical significance (*p* < 0.01) were marked ∗∗. *P* values reaching statistical significance (*p* < 0.05) were marked ∗. (B) Approximation of the sample shape to a cylinder. (C) Real shape of the pure Coll/CMC scaffold.

### 3.3 Swelling Properties

To investigate the swelling properties of scaffolds, the samples were immersed in dPBS (pH 7.4), and weighed at 1, 3, 5, 10, 15, 30, 45, 60, 90, and 120 min. Swelling ratios were calculated according to eq 2. Tukey’s statistical test in Origin was employed to compare the average swelling ratios after 60 min, as this time point was identified as the approximate equilibrium between swelling and degradation. To examine the impact of *in situ* nanoparticle preparation on the swelling properties of the scaffolds, the following samples were analyzed: pure Coll/CMC scaffold (38 ± 5), DOPA-*in situ* prepared sample (43 ± 18), DOPA-*in situ*/Cu sample (36 ± 10), TA-*in situ* prepared sample (42 ± 12), and TA-*in situ*/Cu sample (39 ± 8). Statistical analysis showed no significant differences within the range of standard deviation, indicating similar swelling properties among these samples after 60 min (Figure 6).

**Figure 6:**
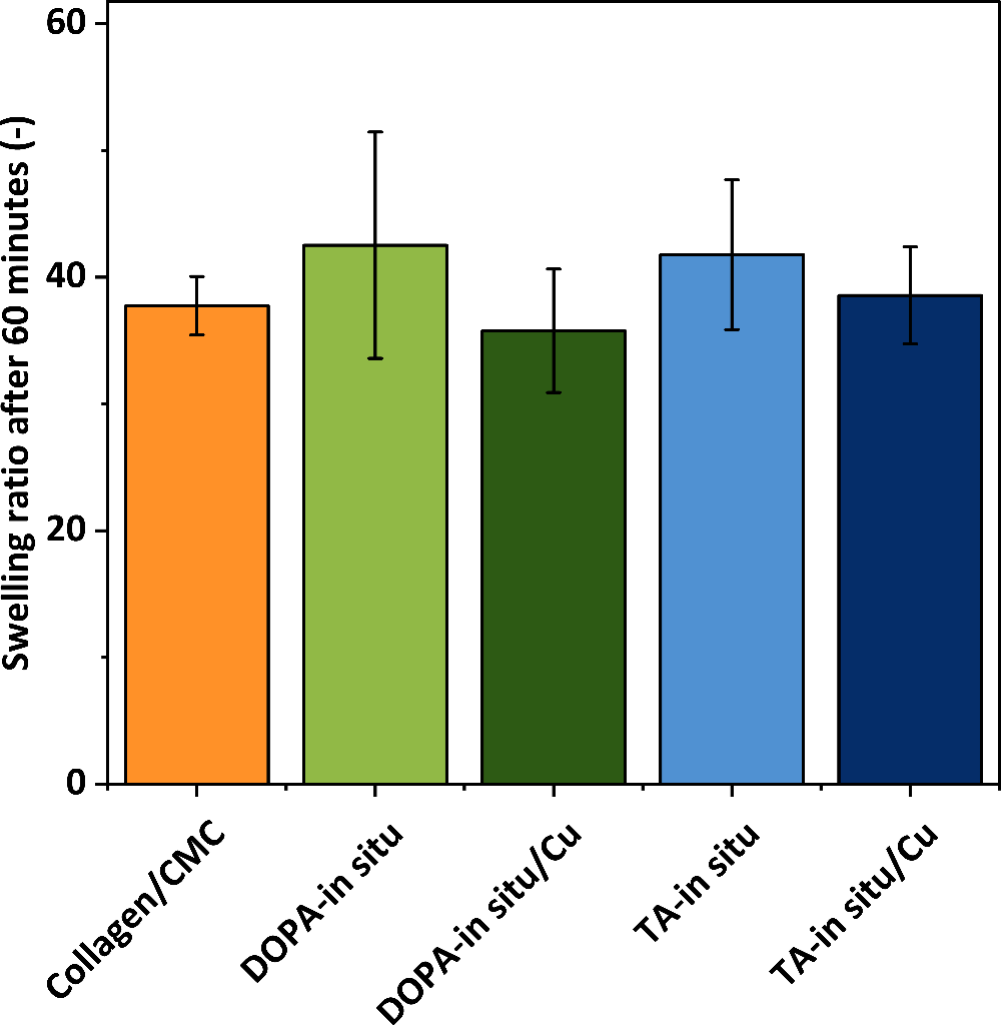
Comparison of swelling ratios of samples. No statistical significances were found, according to Tukey’s statistical test.

### 3.4 Hydrolytic and Enzymatic Stability

The hydrolytic stability of both the pure Coll/CMC scaffold and the Coll/CMC scaffold with DOPA/TA stabilized CuNPs was evaluated in the dPBS buffer. For accelerated enzymatic degradation studies, samples were incubated in 2 mL of collagenase from *Clostridium histolyticum* at 10 CDU/mL in the dPBS (pH 7.4) after a 120 min swelling period.

Upon immersion, all samples initially swelled. As depicted in Figure 7A, the pure Coll/CMC scaffold maintained structural stability in dPBS for approximately 20 h. In the presence of an enzyme, pure Coll/CMC scaffolds remained stable for approximately 24 h. Interaction between CMC and collagen is known to involve hydrogen bonding and electrostatic interaction.^44^ Collagen’s triple-helical structure is highly stable and resistant to enzymatic degradation by most enzymes, except for collagenases. Collagenases, such as those produced by *Clostridium histolyticum*, exhibit high specificity for collagen and cleave the X-Gly bond within the repeating -Gly-Pro-X-Gly-Pro-X- sequence found in the nonpolar regions of the collagen structure.^45^ According to the study by Kanth *et.al*, authors have found out that dialdehyde cellulose (DAC) interacts with collagen via hydrogen bonding or other noncovalent interactions. The authors also found that DAC-treated collagen fibers are resistant to collagenase degradation, likely because DAC protects the active sites on collagen that are typically recognized by the enzyme.^46^ A similar mechanism may occur in the Coll/CMC samples, where CMC forms a protective network around the collagen fibers, effectively shielding them from collagenase cleavage by blocking access to the enzyme’s target sites. As a result, the samples exhibit greater stability in the presence of the enzyme and undergo faster erosion when exposed solely to dPBS, as the enzymatic degradation pathway is hindered.

**Figure 7:**
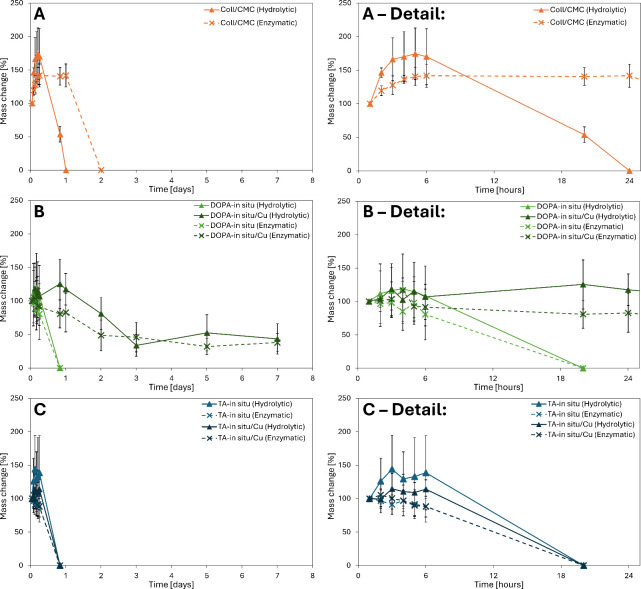
Stability of the prepared samples was evaluated based on the mass change after swelling for 1 h (considered as 100%). (A) Graph illustrating comparison of the hydrolytic and enzymatic stability of the Coll/CMC sample within 8 days and in detail within 24 h. (B) Graph illustrating comparison of the hydrolytic and enzymatic stability of the DOPA-*in situ* samples with and without CuNPs within 8 days and in detail within 24 h. (C) Graph illustrating comparison of the hydrolytic and enzymatic stability of the TA-*in situ* samples with and without CuNPs within 8 days and in detail within 24 h.

As illustrated in Figure 7B, the DOPA-*in situ* sample remained stable for only 6 h in both dPBS and collagenase. Remarkably, the DOPA-*in situ*/Cu sample demonstrated extended stability, remaining intact for up to 168 h (7 days) in both environments. This exceptional stability is likely associated with observations from SEM micrographs, which revealed a deformed inner structure of the collagen scaffold, where fibers appeared “melted-like”. Furthermore, the size of the DOPA-*in situ*/Cu sample was notably smaller, suggesting densification. Additionally, the observed changes in zeta potential within 24 h further support the stabilization of nanoparticles within the scaffold. These findings indicate potential interactions between CuNPs stabilized by DOPA and the collagen matrix. Such interactions may alter the porosity of the scaffold, which could significantly enhance its hydrolytic stability. Polydopamine’s strong adhesive properties likely create robust bonds between CuNPs and the scaffold matrix, including covalent, hydrogen, and π-π interactions, which limit nanoparticle movement. This firm attachment may also explain the observed contraction within the scaffold containing CuNPs stabilized by DOPA, as the polymerization process can introduce slight pulling forces on the matrix due to the formation of these tightly packed bonds.^47,48^

Figure 7C represents the results of the stability evaluation of TA-based samples. The TA-*in situ*, and TA-*in situ*/Cu samples exhibited significantly reduced stability up to 6 h in both environments. Tannic acid offers less consistent stabilization due to its polyphenolic structure, which forms initially stable but ultimately weaker complexes with copper. Over time, the degradation of these polyphenolic aggregates likely leads to the release of more labile copper species, which in turn compromises the stability of the scaffold.^49^

From a practical standpoint, stability for 7 days is considered sufficient, as the CuNPs-loaded collagen foams are intended for topical application and are typically replaced every 3–4 days according to standard clinical wound care practices.^50,51^

### 3.5 Copper Release Measurement

To investigate the release profile of Cu from the Coll/CMC scaffold, UV–vis spectroscopy was performed to construct the absorption and the calibration curve from Cu standards (Supplementary data 1). Initially, the copper from DOPA-*in situ*/Cu and TA-*in situ*/Cu samples was released using LCW 902 Crack Set, and clear solutions were colored to light purple with HI95747–01 Copper LR reagent, to obtain the concentration of Cu incorporated in the scaffold during the synthesis. A lower concentration of copper was obtained for the DOPA-*in situ*/Cu sample (c = 1.1 ± 0.2 mg/L), while a higher copper concentration was observed in the TA-*in situ*/Cu sample (c = 1.5 ± 0.3 mg/L).

Further, the time-dependent release of copper from CuNPs encapsulated by DOPA and CuNPs encapsulated by TA capsules is shown in Figure 8A. Absorption was measured at λ = 560 nm at intervals of 30 min, 1, 2, 4, 8, and 24 h. For the 24-h measurement, the nanoparticles were fully released using the LCW 902 Crack-Set. Copper concentrations were calculated from the linear regression. After 8 h approximately 59 ± 0.2% of copper was released from CuNPs encapsulated by DOPA, while 59 ± 1.2% was released from CuNPs encapsulated by TA.

**Figure 8:**
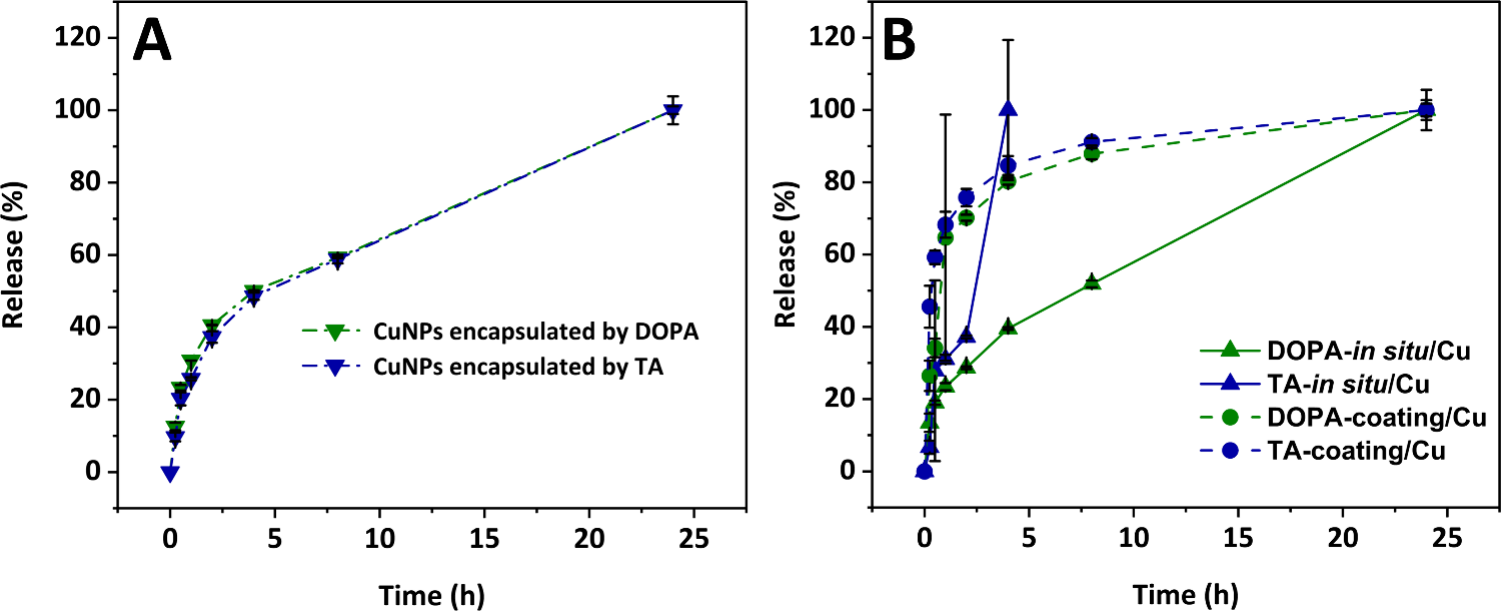
(A) Cumulative release of copper from copper nanoparticles encapsulated in DOPA (green triangles) and TA (blue triangles). (B) Cumulative release of copper nanoparticles from scaffolds with *in situ* prepared Cu NPs encapsulated in DOPA (green triangles) and TA (blue triangles), compared to scaffolds prepared by coating with in advance prepared encapsulated nanoparticles in DOPA (green circles) and TA (blue circles).

The release of copper from Coll/CMC scaffolds, both prepared in situ and applied by coating, is shown in Figure 8B. Absorption measurements were taken at λ = 560 nm at intervals of 30 min, 1, 2, 4, 8, and 24 h. The maximum release (100%) was determined based on the cumulative copper concentrations released over 24 h. For the DOPA-coating/Cu sample, approximately 88 ± 1.5% of copper was released within 8 h. Similarly, the TA-coating/Cu sample showed a release of 91 ± 1.0% in the same period. These results suggest a concentration-dependent, first-order release.

In contrast, the DOPA-*in situ*/Cu sample released only 52 ± 1.0% of copper after 8 h, while the TA-*in situ*/Cu sample degraded before the 8-h mark. The last measurement for the TA-in situ/Cu sample was taken at 4 h, at which point 37 ± 0.4% of copper had been released. These findings indicate that copper release differs significantly between in situ-prepared and coated samples. The more linear release profile observed in *in situ* samples suggests a zero-order release mechanism, which is independent of concentration. This difference may indicate that DOPA and TA are not acting as encapsulating agents, but rather as reducing agents in the nanoparticle synthesis process. Additionally, both TA and DOPA in situ likely facilitate the attachment of copper nanoparticles to collagen fibers via covalent and noncovalent interactions, leading to faster copper release. This rapid release could be advantageous for antibacterial applications. The difference between TA and DOPA in *in situ*-prepared samples is likely due to DOPA’s strong adhesive properties, which allow it to hold nanoparticles more effectively, whereas TA forms weaker interactions, resulting in faster degradation.^52–54^

Release rate constants were determined using MATLAB software. Data fitting was performed based on the Hill equation, and the corresponding results are provided in the Supplementary Data (2–5). The last data point in each graph was excluded from the regression analysis, as it represents the state of 100% release, which occurs due to targeted material degradation.

The constants obtained from the Hill model have a physical significance: the maximum released concentration *V*_max_, the half-release time *K*, and the exponent *n*, which characterizes the release mechanism. The values of these parameters enable the interpretation of material behavior and classification of the studied systems based on their dominant release kinetics.

The *in situ*-DOPA material exhibits diffusion-controlled release, with an exponent *n* approaching 0.5, indicating Fickian diffusion. In contrast, other systems follow predominantly first-order kinetics, with an exponent n approaching 0.89. The *in situ*-DOPA material is driven by standard diffusion and has a relatively high half-release time of 484.3 min. This extended release time suggests that DOPA likely forms an interpenetrating network within the collagen material. This network structure manifests in the mechanical behavior of the material. It has increased the stiffness and brittleness of the cross-linked material after the addition of DOPA. It results in a gradual and prolonged release. Although the release kinetics are slow, nearly 100% of the encapsulated nanoparticles are eventually released over an extended period.

Conversely, the *in situ*-TA system does not form an additional interpenetrating network, leading to significantly faster Cu nanoparticle release. The half-release time is the shortest among all tested samples (20.5 min), with only 44.6% of nanoparticles being released. On the other hand, this material is also more subject to degradation.

When nanoparticles were encapsulated in either DOPA or TA, the release followed first-order kinetics, with differences between these systems arising solely from variations in kinetic rates. *V*_max_ < 100% indicates that the release follows first-order kinetics. In contrast, diffusion-driven release leads to nearly 100% release over a prolonged period.

Nanoparticles encapsulated in DOPA were released relatively quickly, with a half-release time of 2.1 min and a total release of 67.0% of all nanoparticles. In contrast, nanoparticles encapsulated in TA exhibited a slower release, with a half-release time of 205.3 min, but a higher overall release of 89.4% over an extended time. This suggests that in the DOPA system, nanoparticles are more strongly bound within the network structure, whereas in the TA system, release occurs more gradually with higher overall efficiency.

### 3.6 Antibacterial Testing

The antibacterial properties of DOPA-*in situ*/Cu and TA-*in situ*/Cu scaffolds were tested against Gram-negative *E. coli*, Gram-positive *S. aureus*, and moreover, resistant MRSA. The results for *E. coli* are presented in Figure 9A. Compared to the pure Coll/CMC scaffold, the DOPA-*in situ*/Cu samples demonstrated moderate antibacterial activity, which increased with higher concentration of CuNPs in the treated samples. However, the TA-*in situ*/Cu scaffolds did not inhibit bacterial growth compared to the control scaffold. On the contrary, the number of *E. coli* colonies significantly increased at the concentration of 1 mg/mL of CuNPS, suggesting the positive effect of the samples on bacteria survival and metabolism. The results for *S. aureus* (Figure 9B) demonstrate the strongest antibacterial efficiency among all samples, even nanoparticle concentration of 1 μg/mL significantly reduced the bacterial growth (*p* < 0.05). Furthermore, it is noteworthy that the scaffolds were also highly effective against MRSA (Figure 9C). A concentration-dependent effect was observed for both stabilizers (DOPA/TA). Compared to the control scaffold, the bacterial inhibition was higher at 5 μg/mL (*p* < 0.01) than at 1 μg/mL (*p* < 0.05). The primary mode of action described for CuNPs includes oxidative stress induced by ROS, which leads to membrane integrity damage, lipid oxidation or degradation of DNA or proteins.^58^ However, the Gram-negative bacteria possess robust antioxidant defense mechanisms that aid in neutralizing ROS, along with an outer membrane that acts as a barrier against NPs penetration.^59,60^ When using scaffolds for wound healing, their effectiveness against Gram-positive bacteria and their resistant strains is essential, as Staphylococcus species is one of the major contributors to complications in the healing process.^61^

**Figure 9:**
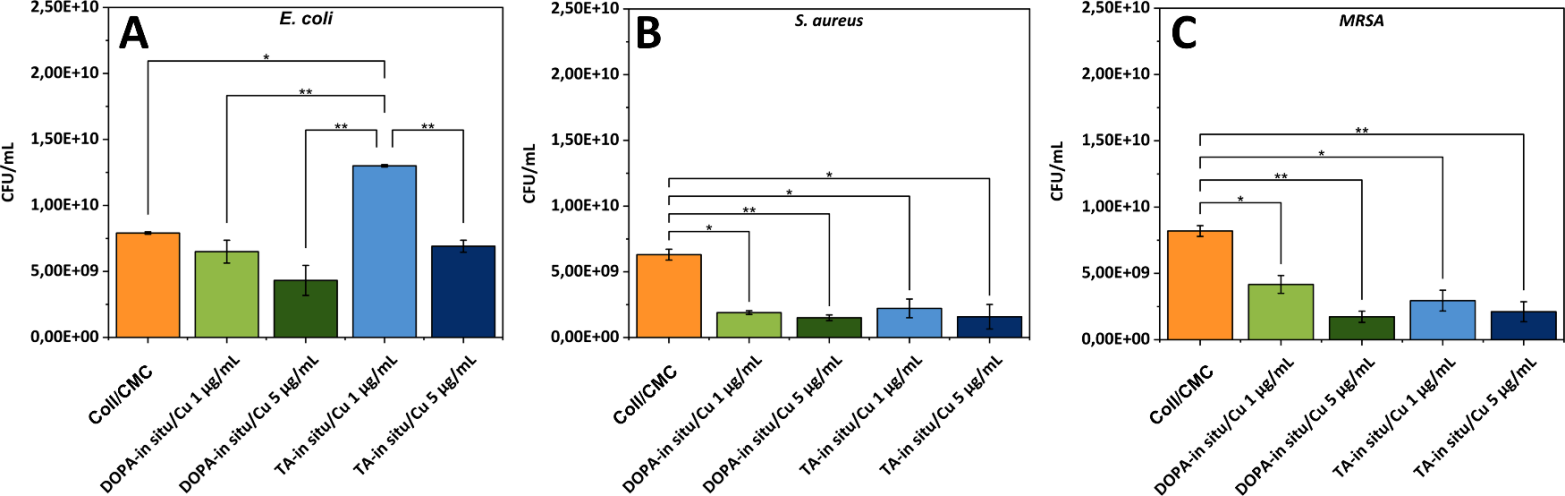
Results of the antibacterial activity of tested samples against (A) *Escherichia coli*, (B) *Staphylococcus aureus*, and (C) methicillin-resistant *S. aureus* (MRSA). *P* values reaching statistical significance (*p* < 0.01) were marked ∗∗; *P* values reaching statistical significance (*p* < 0.05) were marked ∗.

These results suggest that CuNPs-based treatments have the potential to serve as an alternative to commercial antibiotics in the future, helping to prevent the development of bacterial resistance. Copper is safe for topical use in controlled concentrations, particularly in wound dressings where release rates below 10 μg/mL ensure biocompatibility. FDA-approved copper oxide dressings show antimicrobial efficacy and promote healing through angiogenesis and collagen stabilization while maintaining safety.^55,56^ The human body can store approximately 50–120 mg of copper. The average concentration of copper in the blood of an adult man or woman ranges from 63.5–158.9 μg/dcL.^57^ All of these concentrations are much higher than the effective antibacterial concentrations of the samples that were tested.

### 3.7 Cytotoxicity Assay

The extract method evaluating in vitro cytotoxicity of DOPA-*in situ*/Cu and TA-*in situ*/Cu samples was employed to detect any leaching toxic substances from the samples. The results obtained using the quantitative XTT assay are shown in Figure 10. Results suggest that the cytotoxicity levels of all analyzed samples are comparable to the pure Coll/CMC scaffold, indicating no cytotoxicity toward fibroblastic cells. Notably, the sample DOPA-*in situ*/Cu, containing 5 μg/mL of CuNPs even increased cell viability, suggesting a positive effect of copper on cell growth. This finding aligns with previous research indicating that copper, as an essential component of enzymes and proteins involved in cellular metabolism and respiration, supports cellular growth, metabolism, and proliferation.^62,63^

**Figure 10:**
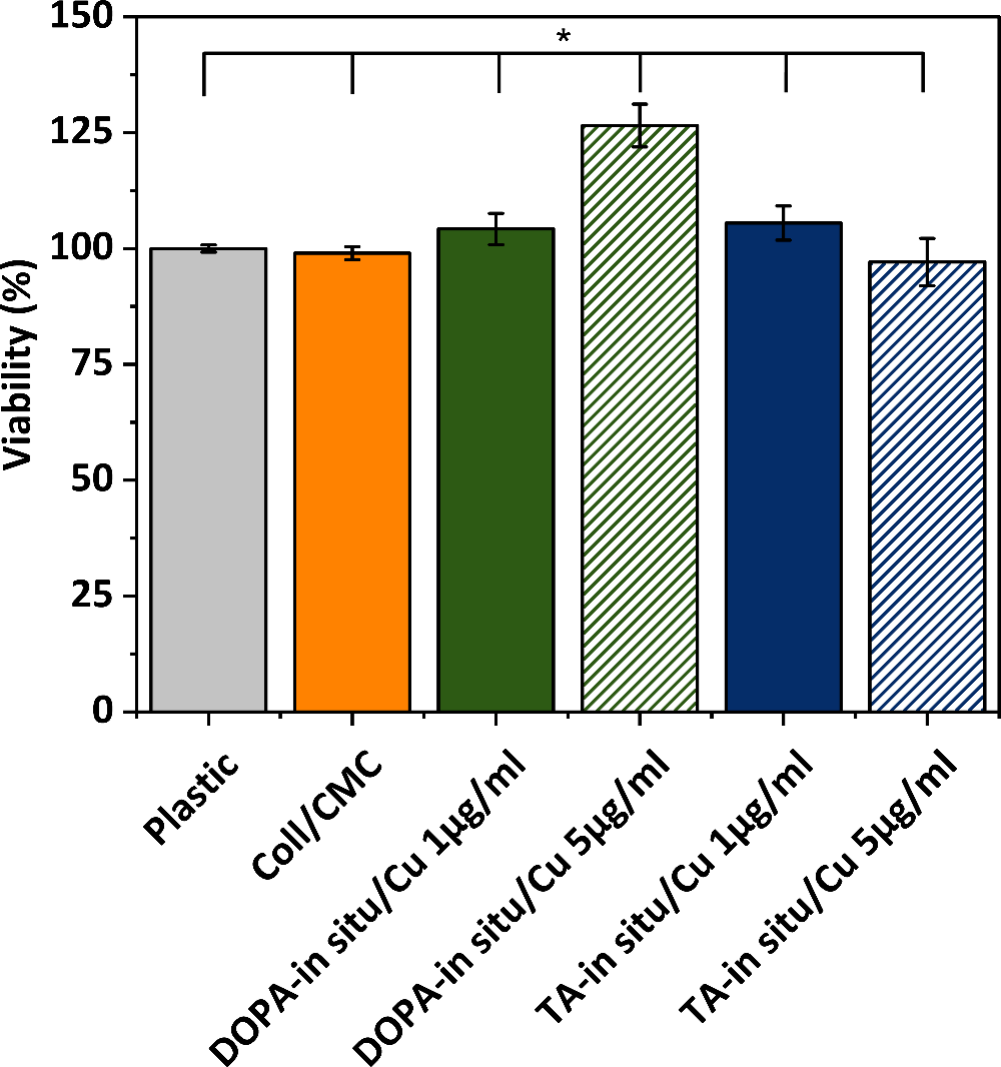
Results of the extraction method for *in* *vitro* cytotoxicity of the scaffolds. The viability was measured using the XTT assay. Statistical significance (∗) was found at α = 0.05.

## 4 Conclusion

This study successfully demonstrated the *in situ* synthesis of CuNPs within Coll/CMC scaffolds, utilizing DOPA and TA as stabilizing and reducing agents inspired by an encapsulation method. The *in situ* method ensured a homogeneous distribution of CuNPs throughout the scaffold, enhancing structural integrity while minimizing localized cytotoxicity.

The incorporation of CuNPs, particularly those stabilized by DOPA, significantly altered the scaffold’s structure, reducing its volume by more than 50%. Notably, DOPA-*in situ*/Cu scaffolds exhibited remarkable enzymatic stability, remaining intact for up to 7 days, which is a substantial improvement over the 20-h stability observed in unmodified Coll/CMC scaffolds.

Copper release kinetics revealed that *in situ*-generated CuNPs were not encapsulated but rather formed through the reductive action of DOPA and TA. The controlled and sustained release observed in DOPA-*in situ*/Cu samples suggests that polydopamine’s strong adhesive properties facilitated CuNP attachment to collagen fibers, stabilizing their release profile. In contrast, TA-*in situ*/Cu scaffolds degraded more rapidly due to weaker interactions.

Biological assays confirmed the enhanced antibacterial activity of scaffolds with *in situ*-generated CuNPs, demonstrating significant efficacy against *S. aureus* and MRSA, and *E. coli* at low copper concentrations while maintaining biocompatibility with 3T3 fibroblasts. These findings highlight the potential of CuNP-based Coll/CMC scaffolds as advanced wound dressings with antibacterial properties and controlled drug release.

Future research should prioritize comprehensive *in vivo* studies to evaluate the biocompatibility, antibacterial efficacy, and wound healing potential of the CuNP scaffolds under physiological conditions. In parallel, a thorough safety assessment, including acute and chronic toxicity, allergenicity, and overall biological safety, will be essential to validate the clinical applicability of these scaffolds. Additionally, further efforts should focus on optimizing their stability and therapeutic efficacy to enhance their performance in real-world biomedical applications.

## Supplementary Material



## Data Availability

All data are available as a data set here: 10.5281/zenodo.15094993. Preprint is available here: 10.5281/zenodo.15181197.

## Notes

Calibration line for determination of copper concentration, the release graphs and calculated kinetic constants for copper nanoparticles encapsulated in DOPA, TA, DOPA *in situ*/Cu scaffold, and TA *in situ*/Cu scaffold, and the calculation of average number of encapsulated particles in the capsule (PDF)

## ABBREVIATIONS

CFU: Colony-Forming Unit
CMC: Carboxymethyl Cellulose
Coll: Collagen
CNR: Contrast-to-Noise Ratio
CT: Computed Tomography
CuNPs: Copper Nanoparticles
DAC: Dialdehyde Cellulose
DLS: Dynamic Light Scattering
DMEM: Dulbecco’s Modified Eagle Medium
DNA: Deoxyribonucleic Acid
DOPA: Dopamine
DS: Degree of Substitution
ECM: Extracellular Matrix
EDC: 1-Ethyl-3-(3-(dimethylamino)­propyl)­carbodiimide
FBS: Fetal Bovine Serum
MRSA: Methicillin-Resistant *Staphylococcus aureus*
NHS: *N*-Hydroxysuccinimide
NP: Nanoparticle
NPs: Nanoparticles
PBS: Phosphate-Buffered Saline
ROS: Reactive Oxygen Species
SEM: Scanning Electron Microscopy
STEM: Scanning Transmission Electron Microscopy
TA: Tannic Acid
UV: Ultraviolet
VIS: Visible Spectrum
XTT: 2,3-Bis­(2-methoxy-4-nitro-5-sulfophenyl)-2*H*-tetrazolium-5-carboxanilide
