# Supplementary material for: In Situ Homogeneous Generation of Copper Nanoparticles in Collagen-Cellulose Freeze-Dried Foams Using Natural Reduction Agents to Enhance Their Stability, Antibacterial Properties, and Cytocompatibility
Source: ACS Omega. 2025 Jul 28;10(35):39799–813. doi: 10.1021/acsomega.5c03661 (PMC12423890; doi:10.1021/acsomega.5c03661)
Supplement: Supplementary file 1 [file ao5c03661_si_001.pdf]

Supporting Information

In Situ Homogeneous Generation of Copper Nanoparticles in Collagen-Cellulose Freeze-Dried Foams Using Natural Reduction Agents to Enhance Their Stability, Antibacterial Properties, and Cytocompatibility

Veronika Polakova[1], Jana Matulova[1], Jana Brtnikova[1], Zdenka Fohlerova[1], Kristyna Smerkova[2], Jozef Kaiser[1, 3], Tomas Zikmund[1], Petra Prochazkova[1], Jan Zidek[1], Lucy Vojtova[1]*

[1]Central European Institute of Technology, Brno University of Technology, Purkynova 123, 612 00, Brno, Czech Republic

[2]Department of Chemistry and Biochemistry, Faculty of AgriSciences, Mendel University in Brno, Zemedelska 1, 613 00 Brno, Czech Republic

[3]Faculty of Mechanical Engineering, Brno University of Technology, Technicka 2, 616 69 Brno, Czech Republic

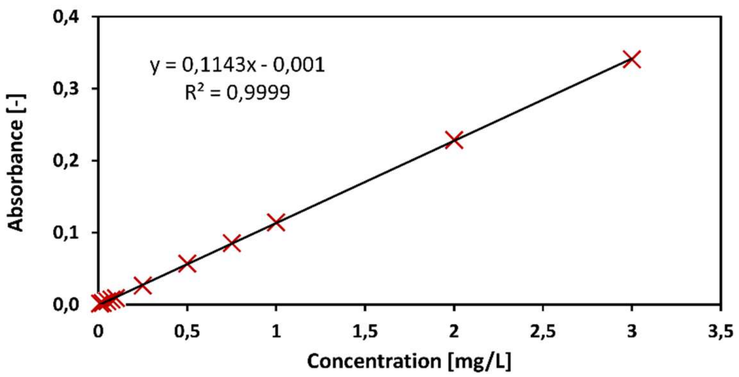

Figure S1: Calibration line for determination of copper concentration.

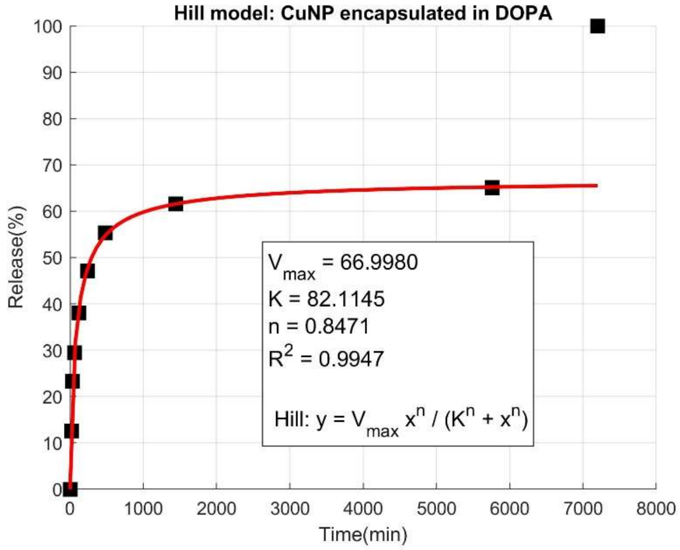

Figure S2: The release graph and calculated kinetic constant for copper nanoparticles encapsulated in DOPA.

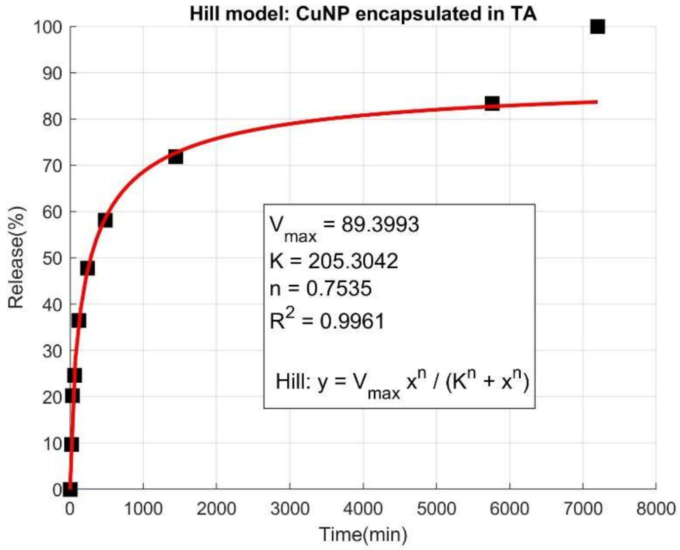

Figure S3: The release graph and calculated kinetic constant for copper nanoparticles encapsulated in TA.

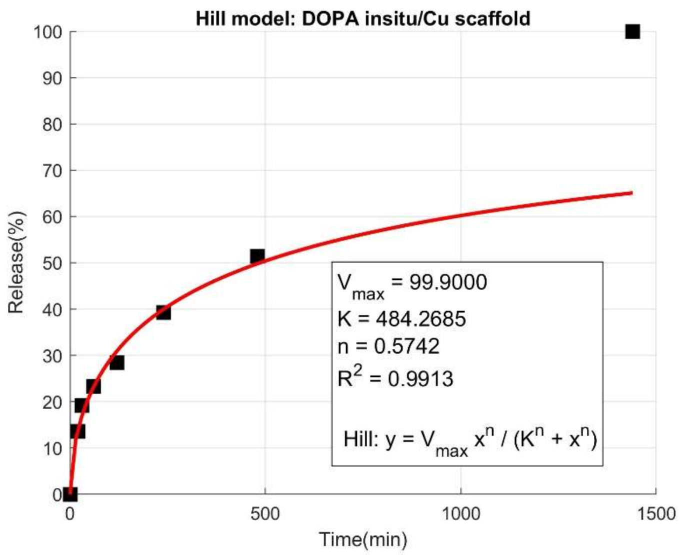

Figure S4: Supplementary data 1: The release graph and calculated kinetic constant for DOPA-in situ/Cu scaffold.

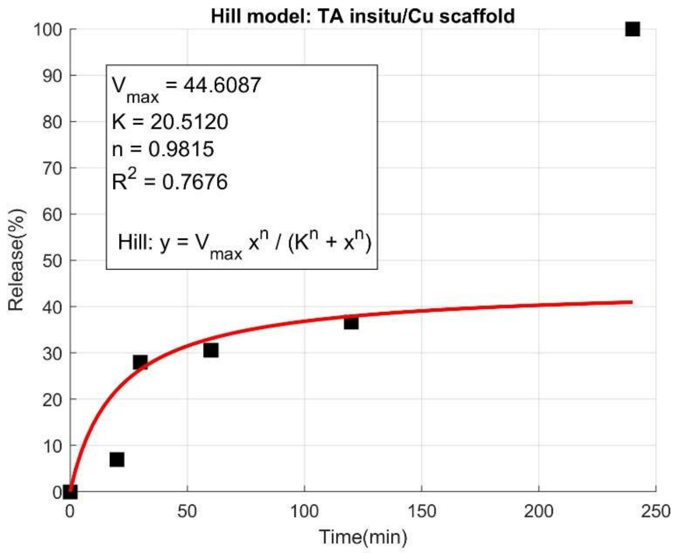

Figure S5: The release graph and calculated kinetic constant for TA-in situ/Cu scaffold.

1. The calculation of average number of encapsulated particles in capsule

The mean diameter of the capsules was obtained from Dynamic Light Scattering (DLS) measurements, while the size of the nanoparticles was determined using Scanning Transmission Electron Microscopy (STEM). Both the capsules and nanoparticles were approximated as spheres, and their volumes were calculated using the following equation:

$$V_c, V_{NP} = \left(\frac{4}{3}\right) \times \pi \times r^3$$

where V_C is the volume of a capsule, V_{NP} is the volume of a nanoparticle, and r is the radius of the respective structure. The theoretical maximum number of nanoparticles that can be encapsulated within a single capsule was then estimated using the formula:

$$N_{NP} = \frac{V_C}{V_{NP}}$$

where N_{NP} represents the maximum number of nanoparticles that could theoretically fit inside one capsule, assuming ideal packing without considering interparticle spacing or structural constraints.
